# Increase in relative skeletal muscle mass over time and its inverse association with metabolic syndrome development: a 7-year retrospective cohort study

**DOI:** 10.1186/s12933-018-0659-2

**Published:** 2018-02-05

**Authors:** Gyuri Kim, Seung-Eun Lee, Ji Eun Jun, You-Bin Lee, Jiyeon Ahn, Ji Cheol Bae, Sang-Man Jin, Kyu Yeon Hur, Jae Hwan Jee, Moon-Kyu Lee, Jae Hyeon Kim

**Affiliations:** 1 Division of Endocrinology and Metabolism, Department of Medicine, Samsung Medical Center, Sungkyunkwan University School of Medicine, Gangnam-gu, Seoul, 06351 South Korea; 2 Division of Endocrinology and Metabolism, Department of Medicine, Samsung Changwon Hospital, Sungkyunkwan University School of Medicine, Changwon, 51524 South Korea; 3 Department of Health Promotion Center, Samsung Medical Center, Sungkyunkwan University School of Medicine, Seoul, 06351 South Korea; 4 Department of Clinical Research Design and Evaluation, SAIHST, Sungkyunkwan University, Seoul, 06351 South Korea

**Keywords:** Metabolic syndrome, Skeletal muscle, Change, Longitudinal study

## Abstract

**Background:**

Skeletal muscle mass was negatively associated with metabolic syndrome prevalence in previous cross-sectional studies. The aim of this study was to investigate the impact of baseline skeletal muscle mass and changes in skeletal muscle mass over time on the development of metabolic syndrome in a large population-based 7-year cohort study.

**Methods:**

A total of 14,830 and 11,639 individuals who underwent health examinations at the Health Promotion Center at Samsung Medical Center, Seoul, Korea were included in the analyses of baseline skeletal muscle mass and those changes from baseline over 1 year, respectively. Skeletal muscle mass was estimated by bioelectrical impedance analysis and was presented as a skeletal muscle mass index (SMI), a body weight-adjusted appendicular skeletal muscle mass value. Using Cox regression models, hazard ratio for developing metabolic syndrome associated with SMI values at baseline or changes of SMI over a year was analyzed.

**Results:**

During 7 years of follow-up, 20.1% of subjects developed metabolic syndrome. Compared to the lowest sex-specific SMI tertile at baseline, the highest sex-specific SMI tertile showed a significant inverse association with metabolic syndrome risk (adjusted hazard ratio [AHR] = 0.61, 95% confidence interval [CI] 0.54–0.68). Furthermore, compared with SMI changes < 0% over a year, multivariate-AHRs for metabolic syndrome development were 0.87 (95% CI 0.78–0.97) for 0–1% changes and 0.67 (0.56–0.79) for > 1% changes in SMI over 1 year after additionally adjusting for baseline SMI and glycometabolic parameters.

**Conclusions:**

An increase in relative skeletal muscle mass over time has a potential preventive effect on developing metabolic syndrome, independently of baseline skeletal muscle mass and glycometabolic parameters.

**Electronic supplementary material:**

The online version of this article (10.1186/s12933-018-0659-2) contains supplementary material, which is available to authorized users.

## Background

Metabolic syndrome is a global health problem along with its individual risk factors, such as central obesity, dyslipidemia, hypertension, and insulin resistance [1]. Estimates indicate that 50 million Americans had metabolic syndrome in 1990 and that number increased to 64 million in 2000. In Asian population, the prevalence of metabolic syndrome grew from 24.9% in 1998 to 31.3% in 2007 in Korea [2–4]. The recent rapid increase in metabolic syndrome prevalence has major socioeconomic implications worldwide due to its significant association with comorbidities, including cardiovascular disease, diabetes, and various cancers, and mortality [5–9].

In terms of body composition, the role of skeletal muscle mass and adiposity, rather than body mass index (BMI), has been the focus of research into risk for metabolic syndrome, particularly in Asian populations, who have relatively increased insulin resistance despite low BMI [10, 11]. Because skeletal muscle is the major site of insulin-mediated glucose utilization (up to 80% in the postprandial state), losses in skeletal muscle mass may lead to metabolic impairments [12]. Furthermore, skeletal muscle is considered to be an endocrine organ because it releases myokines that mediate crosstalk between muscle, adipose tissue, the liver, brain, and other organs in autocrine and paracrine fashions [13].

Recent cross-sectional studies reported that low muscle mass is an important factor for determining metabolic syndrome presence [14–17]. Most previous cross-sectional studies have assessed low muscle mass or muscle mass to adipose tissue ratio of relevance to sarcopenia or sarcopenic obesity, which is an age-related muscle mass loss, to evaluate the relationship with metabolic syndrome, insulin resistance, and diabetes [18]. A further longitudinal study is needed to elucidate the casual relationship between low muscle mass and metabolic syndrome incidence across the full age range of the population, beyond the sarcopenia context. Furthermore, to date, no studies have investigated the relationship between changes in skeletal muscle mass over time and metabolic syndrome development. Therefore, we investigated whether baseline skeletal muscle mass and its changes over time have independent associations with metabolic syndrome development in a large 7-year longitudinal study.

## Methods

### Study population and design

**Fig. 1:**
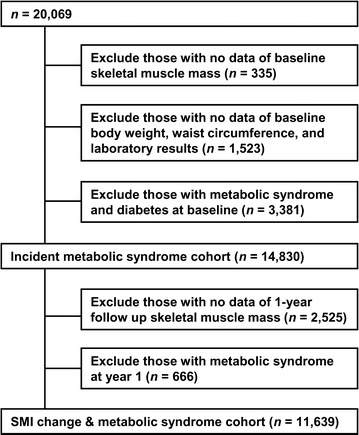
Study flow diagram

In this longitudinal cohort study, we enrolled 20,069 subjects 20 years of age or older who underwent comprehensive health examinations either annually or biennially for four or more follow-up years from August 2006 through August 2013 at the Health Promotion Center at Samsung Medical Center, Seoul, Republic of Korea. We excluded 335 subjects with missing baseline skeletal muscle mass data, 1523 subjects with missing baseline body weight, waist circumference, and laboratory results data, and 3381 subjects who had metabolic syndrome or diabetes at baseline (Fig. 1). A total of 14,830 individuals were included in the analyses of the relationship between skeletal muscle mass at baseline and metabolic syndrome risk. The incidence of metabolic syndrome was defined as the first event during the follow-up and the median follow-up period was 59.5 ± 12.5 months. To investigate the relationship between changes in skeletal muscle mass after 1 year and metabolic syndrome risk, 2525 individuals who were lacking 1-year follow-up data for skeletal muscle mass and 666 individuals with metabolic syndrome at year 1 were further excluded. Finally, a total of 11,639 metabolic-syndrome-free individuals at year 1 were analyzed regarding the association between changes in skeletal muscle mass and metabolic syndrome development. The Institutional Review Board of Samsung Medical Center approved this study’s protocol and written informed consent was obtained from all individuals before their health check-ups.

### Anthropometric and biochemical measurements

Personal and family medical history, smoking status, exercise, medication use, anthropometric data, and laboratory test results were collected during health check-ups. Subjects were categorized with regard to smoking status (never, past smoker, or current smoker). Exercise status was assessed via self-report questionnaires but frequency or time of exercise was not available (none or regular exercise).

Body weight and height were measured and BMI was calculated as kg/m^2^. Obesity was defined according to the criteria for the Asian and Pacific regions (BMI ≥ 25 kg/m^2^) [19]. Waist circumference was examined at the point between the upper iliac crest and the lowest rib after normal expiration. Blood pressure was measured by trained nurses using a mercury sphygmomanometer after at least 5 min of rest in a sitting position. Skeletal muscle mass for each limb (kg), fat mass (kg), and percent fat mass (%) were estimated via bioelectrical impedance analysis (BIA) measurements using a multifrequency BIA device according to the manufacturer’s instructions (InBody 720; Biospace Inc., Seoul, Korea) after an overnight fast. A tetrapolar eight-point tactile electrode system measures impedance at 1, 5, 50, 250, 500 and 1000 kHz. Total body impedance values were calculated by summing the segmental impedance values, and total muscle mass and appendicular skeletal muscle mass were estimated according to manufacturer’s equation. The BIA technique is a valid tool for the assessment of body composition, showing a good correlation with the dual-energy X-ray absorptiometry (DXA) [20, 21]. The skeletal muscle mass index (SMI) was derived by dividing the sum of the appendicular skeletal muscle mass (ASM) in the four limbs (kg) by body weight (kg) × 100 (= total appendicular skeletal muscle mass/body weight × 100) [22–25]. Change in SMI over 1 year from baseline was calculated by subtracting baseline SMI from SMI at the 1-year follow-up visit. Change in SMI over 1 year was analyzed as a continuous variable and as a categorical variable with three groups (< 0, 0–1, and > 1%). In addition, another muscle mass index of ASM divided by BMI (ASM/BMI), which was developed by the National Institutes of Health (NIH) Sarcopenia Project, was also adopted for a sensitivity analysis [26]. Change in ASM/BMI over 1 year as a continuous variable and as a categorical variable with tertiles were assessed.

Laboratory samples were collected after an overnight fast. Plasma total cholesterol, triglyceride, high-density lipoprotein (HDL) cholesterol, low-density lipoprotein (LDL) cholesterol, and creatinine were measured using a Modular D2400 (Roche Diagnostics, Basel, Switzerland). High-sensitivity C-reactive protein (CRP) was measured using the CRP (II) Latax X2 turbidimetric method (Hitachi Corporation, Tokyo, Japan). Plasma glucose and insulin concentrations were measured using the hexokinase method with Bayer Reagent Packs on an automated chemistry analyzer (Advia 1650 Autoanalyzer; Bayer Diagnostics, Leverkusen, Germany) and an immunoradiometric assay (DIAsource Co., Louvain-la-Neuve, Belgium), respectively. HbA_1c_ level was measured by high performance liquid chromatography on an HLC-723G8 automated glycohemoglobin analyzer (TOSOH, Yokkaichi, Japan). homeostasis model assessment of insulin resistance (HOMA-IR) was calculated from the following formula: [fasting plasma insulin (μIU/mL) × fasting plasma glucose (mg/dL)/405] [27, 28]. Estimated glomerular filtration rate (eGFR) was calculated using the modification of diet in renal disease (MDRD) equation [29]. In this study, we defined impaired fasting glucose (IFG) as a fasting glucose level of 100–125 mg/dL or an HbA_1c_ level of 5.7–6.5% without taking antidiabetic medication, and we defined diabetes as fasting glucose level ≥ 126 mg/dL, HbA_1c_ level > 6.5%, or use of antidiabetic medication [30]. Hypertension was defined as blood pressure ≥ 140/90 mmHg or use of antihypertensive medication. Individuals with three or more of the following criteria were defined as having metabolic syndrome according to the revised National Cholesterol Education Program (NCEP) definition: waist circumference ≥ 90 cm in men or ≥ 80 cm in women using the Asia–Pacific abdominal obesity criteria; serum triglycerides ≥ 150 mg/dL or medication use; HDL cholesterol level < 40 mg/dL in men or < 50 mg/dL in women; blood pressure ≥ 130/85 mmHg or antihypertensive medication use; and serum glucose ≥ 100 mg/dL or antidiabetic medication use [31, 32].

### Statistical analysis

All continuous variables are presented as means ± standard deviations (SDs), and categorical variables are expressed as frequencies with percentages. Data was partly extracted from the Clinical Data Warehouse Darwin-C of Samsung Medical Center for this study. Differences were analyzed using analysis of variance (ANOVA) for continuous variables and Chi square tests for categorical variables. The variable changes were determined by calculating the differences between baseline and the 1-year follow-up visit in each subject. An ANCOVA model was used to compare the change in parameters after adjusting for the corresponding baseline levels. Correlations between changes in SMI and changes in glycometabolic parameters were analyzed using Pearson’s correlation. Cumulative event rates for incident metabolic syndrome were estimated by Kaplan–Meier survival curves, and the equalities were compared with the log-rank test. Cox proportional hazard analysis was performed to determine the independent association between either baseline SMI or changes in SMI over 1 year and risk for metabolic syndrome. For multivariate analyses, model 1 was a crude form; age and sex were adjusted for in model 2; model 3 included model-2 adjustments and BMI; model 4 included model-3 adjustments and family history of diabetes, smoking status, regular exercise, eGFR, and CRP concentrations; and model 5 included model-4 adjustments and baseline SMI. When using ASM/BMI index in the multivariate analyses, model 3 included model-2 adjustments and waist circumference instead of BMI, due to relevant multicollinearity. A cubic spline regression model was applied to determine continuous changes in SMI over 1 year and the adjusted hazards ratio for incident metabolic syndrome in Model 5. All covariates in the multivariate models had a variance inflation factor (VIF) < 5.0, which was considered adequate to avoid relevant multicollinearity [33]. Subgroup analyses were conducted according to sex, sex-specific SMI tertile at baseline, family history of diabetes, IFG, smoking status, regular exercise, obesity, > 50 years old, or insulin resistance (HOMA-IR index > 2.5). A *P* value < 0.05 was considered statistically significant. Statistical analyses were performed using SPSS version 23.0 for Windows (SPSS Inc., Chicago, IL, USA).

## Results

### Baseline characteristics of study participants according to sex-specific skeletal muscle mass index tertile

The baseline characteristics of the 14,830 individuals who were included in the baseline SMI analyses are shown, according to their baseline sex-specific SMI tertile (Table 1). Compared to subjects in the lowest baseline SMI tertile, subjects in the middle or highest baseline SMI tertile tended to be younger, less obese, and to have less fat mass, lower blood pressure, lower incidence of IFG, and healthier glycometabolic laboratory values.

**Table 1:** Baseline characteristics of the study subjects according to sex-specific SMI tertile (N = 14,830)

	Lowest tertile (*n* = 4943)	Middle tertile (*n* = 4944)	Highest tertile (*n* = 4943)	*P* value
Skeletal muscle mass index (SMI) (%)	28.6 (2.6)	31.0 (2.3)	33.6 (2.5)	
Men	30.7 (1.1)	32.9 (0.5)	35.4 (1.4)	
Women	25.9 (1.2)	28.4 (0.6)	31.1 (1.4)	
Age (year)	52.7 (8.5)	50.5 (7.5)	49.0 (7.6)	< 0.001
Sex (women)	2118 (42.8)	2118 (42.8)	2118 (42.8)	1.000
Waist circumference (cm)	85.5 (8.3)	81.5 (7.8)	77.3 (7.6)	< 0.001
Body weight (kg)	67.0 (10.8)	64.6 (10.2)	61.7 (9.8)	< 0.001
BMI (kg/m^2^)	25.1 (2.5)	23.4 (2.1)	21.8 (2.1)	< 0.001
Obesity, *n* (%)	2459 (49.8)	1125 (22.8)	321 (6.5)	< 0.001
ASM (kg)	19.3 (4.3)	20.2 (4.3)	20.8 (4.3)	< 0.001
ASM/BMI (m^2^)	0.768 (0.133)	0.856 (0.132)	0.952 (0.145)	< 0.001
Fat mass (kg)	20.0 (4.0)	16.1 (2.7)	12.4 (3.0)	< 0.001
Percent fat mass (%)	30.1 (5.2)	25.2 (4.2)	20.2 (4.3)	< 0.001
Hypertension, *n* (%)	755 (15.3)	477 (9.6)	348 (7.0)	< 0.001
SBP (mmHg)	117.5 (15.2)	114.3 (14.9)	111.6 (14.6)	< 0.001
DBP (mmHg)	72.4 (10.4)	70.9 (10.6)	69.3 (10.5)	< 0.001
Smoking, never/past/current, *n* (%)	2801/1367/775 (56.7/27.7/15.7)	2726/1382/836 (55.1/28.0/16.9)	2751/1329/863 (55.7/26.9/17.5)	0.132
Regular exercise, *n* (%)	829 (16.8)	692 (14.0)	716 (14.5)	< 0.001
Family history of diabetes, *n* (%)	476 (9.6)	544 (11.0)	5.31 (10.7)	0.060
IFG, *n* (%)	627 (12.7)	589 (11.9)	485 (9.8)	< 0.001
HbA_1c_ (%)	5.4 (0.4)	5.3 (0.4)	5.3 (0.4)	< 0.001
Fasting glucose (mg/dL)	90.3 (8.8)	89.6 (8.9)	88.4 (9.0)	< 0.001
Fasting insulin (μIU/mL)^a^	9.65 (3.98)	8.52 (3.22)	7.74 (3.12)	< 0.001
HOMA-IR^a^	2.17 (0.93)	1.90 (0.76)	1.71 (0.73)	< 0.001
eGFR (mL/min/1.73 m^2^)	89.3 (12.9)	88.7 (12.2)	88.5 (12.0)	0.005
Total cholesterol (mg/dL)	200.9 (33.5)	195.8 (31.9)	189.9 (31.6)	< 0.001
Triglycerides (mg/dL)	125.4 (66.6)	117.4 (67.4)	101.7 (61.3)	< 0.001
HDL cholesterol (mg/dL)	56.4 (13.0)	57.2 (13.5)	60.5 (14.6)	< 0.001
LDL cholesterol (mg/dL)	129.4 (29.3)	124.8 (27.8)	117.5 (27.8)	< 0.001
C-reactive protein (mg/L)	0.14 (0.42)	0.11 (0.29)	0.09 (0.27)	< 0.001

Data are presented as mean (standard deviation) or number (percent)

*ASM* appendicular skeletal muscle mass, *BMI* body mass index, *DBP* diastolic blood pressure, *eGFR* estimated glomerular filtration rate, *HDL* high density lipoprotein, *HOMA*-*IR* homeostasis model assessment of insulin resistance, *IFG* impaired fasting glucose, *LDL* low density lipoprotein, *SBP* systolic blood pressure, *SMI* skeletal muscle mass index

^a^A total of 9963 subjects were analyzed due to missing fasting insulin and HOMA-IR values

### Relationship between baseline skeletal muscle mass index and incident metabolic syndrome

**Fig. 2:**
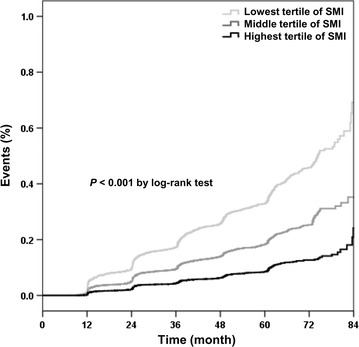
Kaplan–Meier curves for events of incident metabolic syndrome according to baseline sex-specific skeletal muscle mass index tertile

Of the 14,830 subjects, 2983 (20.1%) developed metabolic syndrome during the 7-year follow-up period. The probability of incident metabolic syndrome increased in subjects in the lowest baseline SMI tertile compared with those in the higher tertiles (Fig. 2, *P* < 0.001 by log-rank test). To evaluate the independent association of baseline SMI for developing metabolic syndrome, Cox proportional hazard regression analyses were performed. We found that the highest baseline SMI tertile was significantly associated with a decreased adjusted HR (AHR) for incident metabolic syndrome (0.60, 95% CI 0.54–0.68, *P* < 0.001) compared with the lowest tertile, after adjusting for age, sex, BMI, family history of diabetes, smoking status, regular exercise, eGFR, and CRP, (Model 4, Table 2). The negative association between baseline SMI and metabolic syndrome development remained significant even after an additional adjustment for percent fat mass, but a high VIF of covariates was observed in the analysis (data not shown). Consistent with the results of the inverse relationship between SMI and incident metabolic syndrome, subjects with higher sex-specific ASM/BMI tertiles at baseline had a significant benefit on incident metabolic syndrome, compared to those with the lowest sex-specific ASM/BMI tertile (Additional file 1: Table S1). Table 2 shows that there were stronger reductions in metabolic syndrome incidence in subjects in the higher SMI tertiles compared with the lowest SMI tertile and this pattern was consistent, regardless of sex (Additional file 2: Table S2), family history of diabetes, smoking, exercise status, and age > 50 (All *P*s for interaction > 0.05). On the other hand, the significant benefit was attenuated especially in subjects with obesity or those with insulin resistance (*P*s > 0.05).

**Table 2:** Association between baseline sex-specific SMI tertiles and incidence of metabolic syndrome (Cox model) (N = 14,830)

	Lowest tertile (*n* = 4943)	Middle tertile (*n* = 4944)			Highest tertile (*n* = 4943)			*P* for trend
	Referent	HR	95% CI	*P* value	HR	95% CI	*P* value	
Model 1	1	0.56	0.52, 0.61	< 0.001	0.26	0.24, 0.29	< 0.001	< 0.001
Model 2	1	0.58	0.54, 0.63	< 0.001	0.28	0.25, 0.31	< 0.001	< 0.001
Model 3	1	0.91	0.83, 0.99	0.026	0.63	0.56, 0.70	< 0.001	< 0.001
Model 4	1	0.88	0.81, 0.97	0.006	0.60	0.54, 0.68	< 0.001	< 0.001
Subgroup analyses^a^								
Sex						P for interaction = 0.923		
Men (n = 8476)	1	0.91	0.82, 1.01	0.088	0.65	0.57, 0.75	< 0.001	< 0.001
Women (n = 6354)	1	0.87	0.73, 1.03	0.099	0.56	0.44, 0.70	< 0.001	< 0.001
Family history of diabetes						P for interaction = 0.954		
Absence (n = 13,279)	1	0.91	0.83, 0.99	0.037	0.62	0.55, 0.70	< 0.001	< 0.001
Presence (n = 1551)	1	0.81	0.61, 1.08	0.144	0.47	0.31, 0.70	< 0.001	0.001
Smoking status						P for interaction = 0.314		
Never (n = 8278)	1	0.86	0.75, 0.98	0.022	0.54	0.44, 0.65	< 0.001	< 0.001
Past (n = 4078)	1	0.85	0.73, 0.99	0.039	0.63	0.52, 0.78	< 0.001	< 0.001
Present (n = 2474)	1	0.95	0.80, 1.14	0.603	0.73	0.57, 0.92	0.009	0.023
Exercise						P for interaction = 0.325		
Never (n = 12,593)	1	0.87	0.79, 0.96	0.005	0.62	0.54, 0.70	< 0.001	< 0.001
Regular (n = 2237)	1	0.99	0.79, 1.24	0.940	0.50	0.35, 0.70	< 0.001	< 0.001
Obesity						P for interaction < 0.001		
Absence (n = 10,925)	1	0.77	0.68, 0.87	< 0.001	0.56	0.47, 0.66	< 0.001	< 0.001
Presence (n = 3905)	1	1.04	0.91, 1.18	0.568	0.97	0.85, 1.12	0.698	0.603
						P for interaction = 0.062		
Age ≤ 50 (n = 8017)	1	0.92	0.81, 1.05	0.232	0.60	0.50, 0.72	< 0.001	< 0.001
Age > 50 (n = 6813)	1	0.83	0.74, 0.94	0.002	0.63	0.54, 0.73	< 0.001	< 0.001
IFG						P for interaction = 0.007		
Absence (n = 13,129)	1	0.87	0.79, 0.96	0.006	0.59	0.51, 0.67	< 0.001	< 0.001
Presence (n = 1701)	1	0.91	0.74, 1.13	0.399	0.58	0.44, 0.77	< 0.001	< 0.001
						P for interaction = 0.019		
HOMA-IR^b^ ≤ 2.5 (n = 7957)	1	0.94	0.82, 1.07	0.344	0.71	0.59, 0.84	< 0.001	< 0.001
HOMA-IR > 2.5 (n = 2006)	1	1.14	0.95, 1.37	0.158	0.80	0.64, 1.00	0.053	0.002

Model 1: crude

Model 2: Model 1 + further adjusted for age

Model 3: Model 2 + further adjusted for BMI

Model 4: Model 3 + further adjusted for family history of diabetes, smoking status, regular exercise, eGFR, and CRP

*BMI* body mass index, *CI* confidence interval, *CRP* C-reactive protein, *eGFR* estimated glomerular filtration rate, *HOMA*-*IR* homeostasis model assessment of insulin resistance, *HR* hazard ratio, *IFG* impaired fasting glucose, *SMI* skeletal muscle mass index

^a^Subgroup analyses were adjusted for Model 4

^b^A total of 9963 subjects were analyzed due to missing HOMA-IR values

### Clinical and laboratory characteristics at baseline and at 1-year follow-up according to changes in skeletal muscle mass index over 1 year

We further explored associations between changes in SMI 1 year after baseline and risk for metabolic syndrome among 11,639 individuals. Baseline and 1-year follow-up characteristics of the 11,639 subjects according to SMI changes over 1 year (< 0, 0–1, or > 1%) are shown in Table 3. Subjects with > 1% increase in SMI tended to be younger, female, have lower ASM, lower SMI, and higher fat mass at baseline than those with < 0 or 0–1% (All *P*s < 0.01). Compared with those with < 0 or 0–1% SMI changes, those with SMI increases > 1% were more likely to have a family history of diabetes and higher systolic blood pressure and fasting glucose, and to be IFG at baseline (All *P*s < 0.01). Meaningful differences at year 1 from baseline after adjusting the corresponding baseline values were observed in waist circumference, BMI, ASM, SMI, glycemic parameters, and lipid profile according to SMI change over 1 year (All *P*s < 0.001). As SMI increased at year 1, not only body weight decreased at year 1 but also ASM, skeletal muscle mass itself, increased at year 1 after adjustment for baseline value in subjects with SMI change of 0–1 or > 1% over 1 year (All *P*s < 0.001). We also compared the relationship between SMI changes and changes in glycometabolic parameters for 1-year intervals (Additional file 3: Table S3). We found that SMI changes were significantly correlated with changes in waist circumference (r = − 0.209, *P* < 0.001), body weight (r = − 0.494, *P* < 0.001), ASM (r = 0.588, *P* < 0.001), percent fat mass (r = − 0.834, *P* < 0.001), systolic blood pressure (SBP) (r = − 0.124, *P* < 0.001), triglycerides (r = − 0.126, *P* < 0.001), and HDL cholesterol (r = 0.074, *P* < 0.001). Significant inverse correlations were present between SMI increases and changes in glycemic indices, including ΔHbA_1c_ (r = − 0.063, *P* < 0.001), Δfasting glucose (r = − 0.094, *P* < 0.001), and ΔHOMA-IR (r = − 0.111, *P* < 0.001). However, changes in CRP were not significantly correlated with changes in SMI.

**Table 3:** Characteristics of the study subjects according to changes in SMI over one year (N = 11,639)

	Group 1 (< 0%) (*n* =6525)		Group 2 (0–1%) (*n* = 3734)		Group 3 (> 1%) (*n* = 1380)				
SMI 1-year increases (%)	− 0.80 (0.66)		0.43 (0.28)		1.65 (0.80)				

	Baseline	1-year follow up	Baseline	1-year follow up	Baseline	1-year follow up	*P* value*	*P* value^†^	*P* value^‡^
Age (year)	51.1 (7.9)	52.2 (7.9)	50.5 (7.8)	51.6 (7.8)	49.7 (8.0)	50.8 (8.0)	< 0.001	< 0.001	0.001
Sex (women)	2444 (37.5)	–	1724 (46.2)	–	725 (52.5)	–	< 0.001	–	–
Waist circumference (cm)	81.4 (8.2)	82.2 (8.2)	80.9 (8.6)	80.9 (8.3)	80.2 (8.6)	79.0 (8.1)	< 0.001	< 0.001	< 0.001
Body weight (kg)	64.5 (10.1)	65.2 (10.1)	63.8 (10.5)	63.4 (10.4)	63.1 (10.5)	61.4 (10.0)	< 0.001	< 0.001	< 0.001
BMI (kg/m^2^)	23.3 (2.5)	23.6 (2.5)	23.3 (2.6)	23.1 (2.6)	23.2 (2.7)	22.5 (2.5)	0.750	< 0.001	< 0.001
Obesity, *n* (%)	1527 (23.4)	1817 (27.9)	898 (24.1)	800 (21.4)	347 (25.1)	220 (15.9)	0.355	< 0.001	< 0.001
ASM (kg)	20.5 (4.2)	20.2 (4.2)	19.7 (4.3)	19.9 (4.3)	19.2 (4.3)	19.7 (4.3)	< 0.001	< 0.001	< 0.001
Fat mass (kg)	15.4 (4.4)	16.7 (4.4)	16.4 (4.5)	15.8 (4.3)	16.7 (4.5)	14.3 (4.1)	< 0.001	< 0.001	< 0.001
Percent fat mass (%)	24.0 (6.0)	25.7 (5.9)	25.7 (6.0)	25.0 (5.9)	26.6 (6.1)	23.4 (5.9)	< 0.001	< 0.001	< 0.001
SMI (%)	31.7 (3.1)	30.9 (3.1)	30.7 (3.1)	31.2 (3.1)	30.3 (3.2)	31.9 (3.2)	< 0.001	< 0.001	< 0.001
ASM/BMI (m^2^)	0.881 (0.155)	0.857 (0.151)	0.846 (0.153)	0.859 (0.154)	0.826 (0.156)	0.875 (0.160)	< 0.001	< 0.001	< 0.001
Hypertension, *n* (%)	627 (9.6)	715 (11.0)	333 (8.9)	356 (9.5)	119 (8.6)	130 (9.4)	0.345	0.037	0.035
SBP (mmHg)	113.7 (14.9)	117.8 (15.3)	114.4 (15.1)	116.0 (15.5)	114.7 (15.5)	114.4 (15.9)	0.017	< 0.001	< 0.001
DBP (mmHg)	70.3 (10.3)	74.6 (10.8)	71.0 (10.7)	73.0 (11.1)	71.0 (11.1)	71.6 (11.4)	0.001	< 0.001	< 0.001
Smoking, never/past/current, *n* (%)	3402/1944/1179 (52.1/29.8/18.1)	–	2209/994/531 (59.2/26.6/14.2)	–	869/319/192 (63.0/23.1/13.9)	–	< 0.001	–	–
Regular exercise, *n* (%)	829 (12.7)	–	573 (15.4)	–	236 (17.1)	–	< 0.001	–	–
Family history of diabetes, *n* (%)	590 (9.0)	–	417 (11.2)	–	156 (11.3)	–	0.001	–	–
IFG, *n* (%)	681 (10.4)	872 (13.4)	418 (11.2)	451 (12.1)	172 (12.5)	150 (10.9)	0.073	0.018	< 0.001
Diabetes, *n* (%)	–	91 (1.4)	–	32 (0.9)	–	7 (0.5)	–	0.003	–
HbA_1c_ (%)	5.3 (0.4)	5.4 (0.4)	5.3 (0.4)	5.4 (0.4)|	5.3 (0.5)	5.4 (0.4)	0.098	0.070	< 0.001
Fasting glucose (mg/dL)	89.0 (8.7)	91.2 (9.6)	89.2 (8.7)	90.3 (9.1)	89.4 (9.4)	89.5 (8.9)	0.168	< 0.001	< 0.001
Fasting insulin^a^ (μIU/mL)	8.51 (3.38)	8.29 (3.56)	8.62 (3.41)	7.83 (3.54)	8.47 (3.60)	7.29 (3.58)	0.379	< 0.001	< 0.001
HOMA-IR^a^	1.89 (0.80)	1.89 (0.89)	1.92 (0.80)	1.76 (0.86)	1.90 (0.86)	1.63 (0.88)	0.249	< 0.001	< 0.001
eGFR (mL/min/1.73m^2^)	88.5 (12.1)	88.1 (12.6)	88.8 (12.3)	88.4 (13.1)	89.5 (13.4)	88.7 (14.2)	0.042	0.299	0.496
Total cholesterol (mg/dL)	195.0 (31.9)	199.1 (32.8)	196.9 (33.2)	197.1 (32.2)	196.0 (33.7)	193.9 (32.5)	0.018	< 0.001	< 0.001
Triglycerides (mg/dL)	110.8 (60.2)	115.0 (58.8)	114.1 (69.9)	107.9 (56.1)	109.9 (55.6)	97.8 (45.3)	0.028	< 0.001	< 0.001
HDL cholesterol (mg/dL)	58.3 (13.6)	56.4 (13.5)	58.8 (14.0)	57.5 (13.8)	59.4 (14.3)	59.3 (13.9)	0.016	< 0.001	< 0.001
LDL cholesterol (mg/dL)	123.2 (28.0)	124.2 (27.8)	124.6 (29.1)	121.2 (28.2)	123.9 (29.4)	116.5 (28.0)	0.043	< 0.001	< 0.001
C-reactive protein (mg/L)	0.11 (0.36)	0.11 (0.32)	0.11 (0.27)	0.10 (0.24)	0.10 (0.19)	0.09 (0.24)	0.144	0.069	0.125

Data are presented as mean (standard deviation) or number (percent)

*ASM* appendicular skeletal muscle mass, *BMI* body mass index, *DBP* diastolic blood pressure, *eGFR* estimated glomerular filtration rate, *HDL* high density lipoprotein, *HOMA-IR* homeostasis model assessment of insulin resistance, *IFG* impaired fasting glucose, *LDL* low density lipoprotein, *SBP* systolic blood pressure, *SMI* skeletal muscle mass index

* Analysis of variance (ANOVA) for continuous variables and chi-square tests for categorical variables at baseline

^†^Analysis of variance (ANOVA) for continuous variables and chi-square tests for categorical variables at 1-year follow up

^‡^An ANCOVA model for comparing changes from baseline at year 1 after the adjustment for corresponding baseline values

^a^A total of 9963 subjects and 7701 subjects were analyzed due to missing fasting insulin and HOMA-IR values at baseline and at year 1, respectively

### Relationship between increase in skeletal muscle mass index over 1 year and incident metabolic syndrome

**Fig. 3:**
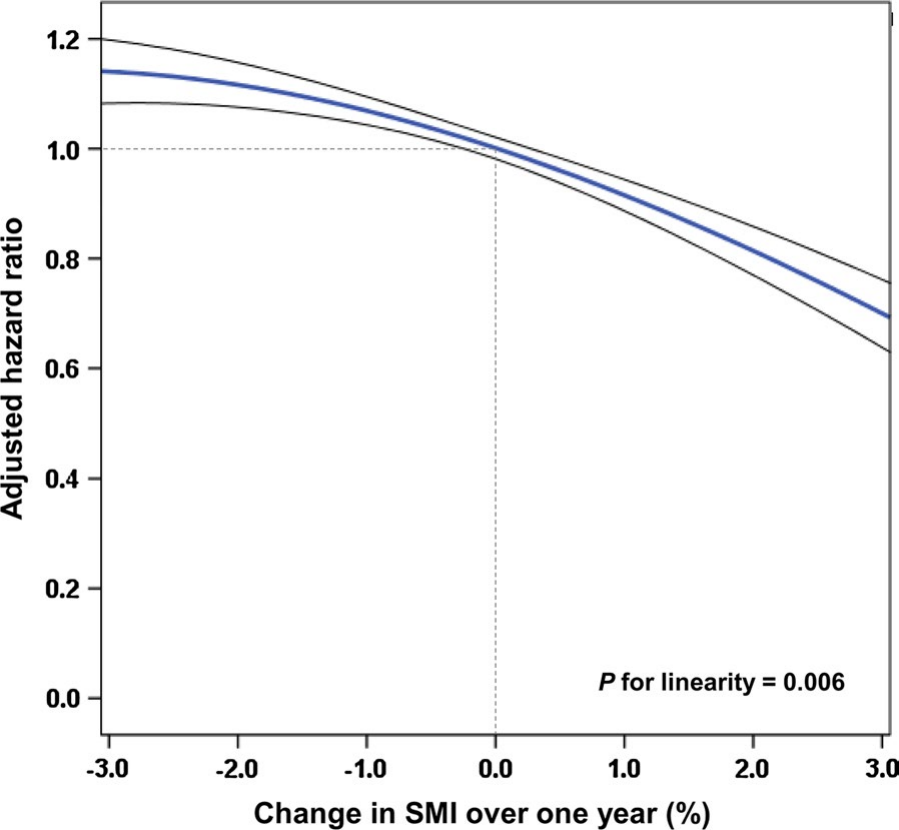
Adjusted HR for incident metabolic syndrome according to changes in SMI 1 year after baseline. The data shown are from cubic splines and 95% CIs are also presented. Adjusted HRs are from Cox proportional-hazards models after adjusting for age, sex, body mass index, family history of diabetes, smoking status, exercise, C-reactive protein concentrations, and SMI at baseline. *CI* confidence interval, *HR* hazard ratio, *SMI* skeletal muscle mass index

Cox proportional hazard analysis was performed to investigate the independent risk of SMI change over 1 year for developing metabolic syndrome. Changes in SMI over 1 year as a continuous variable had a strong inverse association with metabolic syndrome development, after adjusting for several glycometabolic parameters and baseline SMI (AHR = 0.89, 95% CI 0.85–0.94, *P* < 0.001; Model 5; Additional file 4: Table S4). Additionally, we observed a clearly negative linear relationship between SMI change over 1 year and risk for metabolic syndrome in the cubic spline model (*P* for linearity = 0.006; Model 5; Fig. 3).

As shown in Table 4, individuals whose SMI increases were > 1% over a year had AHRs of 0.71 (95% CI 0.59–0.84, *P* < 0.001), for developing metabolic syndrome compared with individuals whose SMI changes were < 0% after adjusting for age, sex, BMI, family history of diabetes, smoking, exercise, and CRP levels, (Model 4). Furthermore, when baseline SMI was also adjusted for, a significant association between SMI increase over 1 year and low risk for metabolic syndrome resulted in AHRs of 0.67 (95% CI 0.56–0.79, *P* < 0.001) and 0.87 (95% CI 0.78–0.97, *P* = 0.010), respectively, among individuals whose SMI increased > 1 and 0–1% versus individuals whose SMI changed < 0% (Model 5). The significant inverse association between change in SMI over 1 year and incident metabolic syndrome was consistent even after additionally adjusting for percent fat mass, which itself was not independently associated with metabolic syndrome in multivariate analyses (data not shown). Subjects with the highest tertile of change in ASM/BMI index over 1 year also showed the beneficial effect on incident metabolic syndrome compared to those with the lowest tertile of change in ASM/BMI, after the adjustment for multiple covariates and baseline ASM/BMI index (Additional file 5: Table S5). Change in ASM/BMI index over 1 year as a continuous variable also had a significant inverse association with metabolic syndrome development (Additional file 6: Table S6). In the subgroup analyses, there was a consistent pattern of benefit of increase in SMI over 1 year on the risk of metabolic syndrome, regardless of sex, sex-specific SMI tertile at baseline, smoking, obesity, age, IFG, and insulin resistance (Table 4; All *P*s for interaction > 0.05). However, the significant association was attenuated in women (Additional file 7: Table S7), subjects in the highest tertile of sex-specific SMI at baseline, subjects with IFG, and with insulin resistance (Table 4; *P*s for trend > 0.05).

**Table 4:** Association between change in SMI over 1 year and incidence of metabolic syndrome (Cox model) (N = 11,639)

	Group 1 (< 0%) (*n* = 6525)	Group 2 (0–1%) (*n* = 3734)			Group 3 (> 1%) (*n* = 1380)			*P* for trend
	Referent	HR	95% CI	*P* value	HR	95% CI	*P* value	
Model 1	1	0.91	0.82, 1.01	0.080	0.72	0.61, 0.86	< 0.001	< 0.001
Model 2	1	0.94	0.85, 1.05	0.282	0.77	0.65, 0.92	0.004	0.013
Model 3	1	0.90	0.81, 1.01	0.066	0.71	0.59, 0.84	< 0.001	< 0.001
Model 4	1	0.91	0.82, 1.01	0.073	0.71	0.59, 0.84	< 0.001	< 0.001
Model 5	1	0.87	0.78, 0.97	0.010	0.67	0.56, 0.79	< 0.001	< 0.001
Subgroup analyses^a^								
Sex						P for interaction = 0.506		
Men (n = 6746)	1	0.83	0.73, 0.94	0.005	0.63	0.50, 0.79	< 0.001	< 0.001
Women (n = 4893)	1	0.95	0.79, 1.16	0.631	0.78	0.58, 1.04	0.085	0.226
Baseline sex-specific SMI						P for interaction = 0.588		
Lowest tertile (n = 3879)	1	0.81	0.70, 0.93	0.004	0.67	0.53, 0.83	< 0.001	< 0.001
Middle tertile (n = 3880)	1	0.98	0.81, 1.18	0.825	0.64	0.45, 0.90	0.011	0.039
Highest tertile (n = 3880)	1	0.88	0.66, 1.18	0.397	0.67	0.40, 1.15	0.145	0.282
Smoking status						P for interaction = 0.204		
Never (n = 6480)	1	0.89	0.76, 1.04	0.132	0.74	0.58, 0.94	0.014	0.032
Past (n = 3257)	1	0.76	0.63, 0.93	0.007	0.71	0.51, 0.99	0.045	0.008
Present (n = 1902)	1	0.99	0.79, 1.24	0.941	0.48	0.31, 0.73	0.001	0.003
Obesity						P for interaction = 0.092		
Absence (n = 8867)	1	0.89	0.76, 1.03	0.109	0.77	0.61, 0.98	0.031	0.050
Presence (n = 2772)	1	0.85	0.73, 1.00	0.047	0.56	0.43, 0.74	< 0.001	< 0.001
						P for interaction = 0.351		
Age ≤ 50 (n = 6326)	1	0.94	0.81, 1.10	0.462	0.70	0.55, 0.90	0.005	0.019
Age > 50 (n = 5313)	1	0.80	0.69, 0.93	0.003	0.62	0.48, 0.81	< 0.001	< 0.001
IFG						P for interaction = 0.456		
Absence (n = 10,368)	1	0.87	0.77, 0.98	0.020	0.63	0.51, 0.77	< 0.001	< 0.001
Presence (n = 1271)	1	0.79	0.60, 1.05	0.106	0.75	0.50, 1.12	0.154	0.160
						P for interaction = 0.631		
HOMA-IR^b^ ≤ 2.5 (n = 6254)	1	0.83	0.71, 0.97	0.023	0.63	0.49, 0.83	0.001	0.001
HOMA-IR > 2.5 (n = 1447)	1	0.87	0.69, 1.10	0.242	0.77	0.54, 1.09	0.136	0.233

Model 1: crude

Model 2: Model 1 + further adjusted for sex and age

Model 3: Model 2 + further adjusted for BMI

Model 4: Model 3 + further adjusted for family history of diabetes, smoking status, regular exercise, eGFR, and CRP

Model 5: Model 4 + further adjusted for baseline SMI

*BMI* body mass index, *CI* confidence interval, *CRP* C-reactive protein, *eGFR* estimated glomerular filtration rate, *HDL* high density lipoprotein, *HOMA*-*IR* homeostasis model assessment of insulin resistance, *HR* hazard ratio, *IFG* impaired fasting glucose, *SMI* skeletal muscle mass index

^a^Subgroup analyses were adjusted for Model 5

^b^A total of 7701 subjects were analyzed due to missing HOMA-IR values

## Discussion

This is the first study to evaluate the relationship between changes in SMI over time and risk for metabolic syndrome in a large 7-year retrospective cohort study. Herein, we show that, even after adjusting for glycometabolic parameters and baseline SMI, there was a significant decrease in the risk of metabolic syndrome by 23% in subjects whose SMI increased > 1% over a year from baseline versus those whose SMI changed < 0%.

### Change in relative skeletal muscle mass and obesity

Previous studies revealed that low skeletal muscle mass was significantly associated with metabolic syndrome in cross-sectional studies [14–17]. Furthermore, low muscle mass was also found to be a risk factor for metabolic syndrome in non-obese subjects but not in obese subjects [16, 34]. Consistent with these findings, we found an inverse association between baseline SMI and metabolic syndrome development in a 7-year longitudinal follow-up study; these findings were consistently observed in non-obese subjects, but not in obese subjects. However, we showed that an increase in relative muscle mass over a single year was significantly associated with low risk of metabolic syndrome even in obese people who may have a high cardiometabolic risk [35], suggesting that an increase in relative skeletal muscle mass is a potent preventive parameter for metabolic syndrome.

There have been various representative methods to estimate relative skeletal muscle mass, using height squared (ASM/height^2^), weight (ASM/body weight (Wt) = SMI), and BMI (ASM/BMI) to adjust body size [22, 26, 36], because ASM is fundamentally correlated with body size [37]. In the present study, we used ASM/Wt (SMI) for assessing relative skeletal muscle mass because a previous study proposed that sarcopenia defined as ASM/Wt was more closely associated with metabolic parameters than sarcopenia defined by ASM/height^2^ [38]. Also, we investigated the change of relative skeletal muscle over time. In terms of change in relative muscle mass over 1 year, a change of SMI between baseline and year 1 could be easily assessed as a percent change by subtracting baseline SMI from SMI at year 1. From a practical perspective, using SMI may be a simple and convenient approach with which laypersons are able to easily assess change in their body composition. In a similar context, several studies have reported an annual loss of approximately 1–2% of lean muscle mass after about age 50 [39–42]. In the present study, as a continuous variable, there was a significant decreased risk of metabolic syndrome by 11% per percent increase in SMI over a year, after adjusting for baseline SMI and glycometabolic parameters. In line with this, a SMI change 0–1 or > 1% over 1 year versus < 0% may have the clinical implication suggesting that an increase in relative skeletal muscle mass is a potent preventive parameter for metabolic syndrome. However, there might be concerns regarding dependence of body weight on SMI when SMI changes. Therefore, we analyzed the change of body composition and glycometabolic parameters between baseline and year 1 after the adjustment for their corresponding values and found that people having an increase in SMI over a year tended to have decreased body weight and increased ASM over a year. Also, a change in SMI was negatively related with body weight, while positively related with ASM. Moreover, we additionally adopted another ASM/BMI index, which was well correlated with cardiometabolic risk factors than when using ASM/ht^2^ [43], for assessing relative skeletal muscle mass for a sensitivity analysis. Consistent with the results using SMI, ASM/BMI index also presented beneficial effects of baseline relative muscle mass and its change over 1 year on incident metabolic syndrome.

### Change in relative skeletal muscle mass and age

Among our study population between 20 and 80 years old, we found a significant inverse association between SMI or SMI changes and risk for metabolic syndrome and, thus, this relationship was not limited to the elder population, e.g., as with age-related sarcopenia; rather this risk is present for the entire age range, indicating the significant clinical importance of relative skeletal muscle mass and its increase, even for younger patients.

### Possible pathophysiological mechanism between muscle mass and metabolic syndrome

There are several possible mechanisms underlying the association between muscle mass loss and risk for metabolic disease. Skeletal muscle is considered as the major site of postprandial glucose utilization. Previously, tissue-specific knockouts of glucose transporter (GLUT) 4 in muscle exhibited severely impaired glucose tolerance and hyperinsulinemia [44], and mice with a knockout of the insulin receptor in muscle revealed increased triglycerides and free fatty acids [45]. Furthermore, because skeletal muscle secretes various myokines, including irisin and interleukin-6 (IL-6), muscle tissue has become increasingly regarded as another endocrine regulator of metabolism [46–48]. Irisin, which is induced by physical activity and peroxisome proliferator-activated receptor-γ coactivator 1α (PGC1α), is a novel hormone implicated in glucose and lipid metabolism [49]. Overexpression of the fibronectin type III domain-containing protein 5 (FNDC5) gene, a precursor of irisin, resulted in adipose tissue browning, increase in oxygen consumption, amelioration of glucose tolerance and hyperinsulinemia, and reduction of obesity in mice [50, 51]. In humans, Park et al. revealed a compensatory increase of irisin in subjects with metabolic syndrome [52], while Kurdiova et al. reported that circulating irisin was negatively associated with fasting glucose concentration, area under the glycemic curve, and waist circumference, and was positively associated with physical activity level [53]. Additionally, *Fndc5* gene expression in human muscle showed positive associations with physical activity and muscle mass. Therefore, large relative muscle mass may be related to efficient glucose uptake and lipid metabolism with high levels of favorable myokines. However, the effect of SMI changes on metabolic syndrome development had not been previously studied. Herein, we also showed that SMI changes were negatively correlated with changes in waist circumference, SBP, HOMA-IR, and concentrations of HbA_1c_, fasting glucose, triglycerides, and LDL cholesterol, but not significantly so with CRP levels. In all these possible pathways, low relative muscle mass may implicate metabolic impairment, which should be taken into account for subjects with low relative muscle mass, including patients who are not obese. Our data indicate increases in relative muscle mass may play a significant role in preventing metabolic syndrome, beyond relative muscle mass at baseline and well-known risk factors. Further studies on potential protective mechanisms underlying this association are needed.

This study has several strengths. First, using a large 7-year cohort study, we investigated SMI changes over a year and we estimated the association between SMI changes developing metabolic syndrome. Our large longitudinal sample strengthens the statistical reliability of our analyses. Second, we reported SMI in numerical values that we estimated using direct segmental multi-frequency BIA analysis, which was valid for building excellent agreement in segmental body composition measurements, particularly for quantifying lean body mass [20, 21]. Third, we demonstrated the significant benefits of SMI increases over 1 year after adjusting for possible confounding glycometabolic parameters and baseline SMI. Moreover, our results from detailed subgroup analyses provided robust evidence of the association between SMI increases and lower risk of metabolic syndrome, independently of obesity or insulin resistance. We also evaluated the correlation between SMI changes and changes in important glycometabolic parameters and CRP levels, a systemic inflammatory marker.

### Limitations

However, our study also has some limitations. First, we could only assess changes in SMI from baseline to year 1. Further investigations measuring longitudinal SMI changes until metabolic syndrome development would be useful. Second, exercise status was not evaluated by specific type, frequency, duration, or intensity, all of which could contribute to changes in relative skeletal muscle mass [54]. Third, data regarding nutritional supplements, concentrations of various myokines, and blood testosterone in male subjects, which could also affect skeletal muscle mass, were not available. Also, although low muscle strength was also reported to be associated with all-cause mortality, independently of muscle mass [55], skeletal muscle strength was not available in the present study. Finally, this study was based on a sample of Korean individuals who participated in health check-up examinations, which may limit the ability to generalize our results to other settings or other ethnicities.

## Conclusions

In conclusion, increases in relative skeletal muscle mass might protect against metabolic syndrome after adjustment for baseline relative skeletal muscle mass and glycometabolic parameters. The strong benefit of relative skeletal muscle mass increases for protecting against metabolic syndrome was present, particularly in men, and subjects who were obese. Considering the increasing rate of obesity worldwide and the deep relationship with various comorbidities with metabolic syndrome, management of relative skeletal muscle mass may contribute to potential prevention of metabolic syndrome.

## Additional files

**Additional file 1: Table S1.** Association between baseline sex-specific ASM/BMI tertiles and incidence of metabolic syndrome (Cox model) (N = 14,830).

**Additional file 2: Table S2.** Association between baseline sex-specific SMI tertiles and incidence of metabolic syndrome in men (N = 8476) and women (N = 6354).

**Additional file 3: Table S3.** Correlations between changes of skeletal muscle mass index and glycometabolic parameters.

**Additional file 4: Table S4.** Association between continuous variable of change in skeletal muscle mass index from baseline to year 1 and incidence of metabolic syndrome (Cox model).

**Additional file 5: Table S5.** Association between change in ASM/BMI index over 1 year and incidence of metabolic syndrome (Cox model) (N = 11,639).

**Additional file 6: Table S6.** Association between continuous variable of change in ASM/BMI index from baseline to year 1 and incidence of metabolic syndrome (Cox model).

**Additional file 7: Table S7.** Association between change in SMI over 1 year and incidence of metabolic syndrome in men (N = 6746) and women (N = 4893).

## Abbreviations

ANOVA: analysis of variance
ASM: appendicular skeletal muscle mass
BIA: bioelectrical impedance analysis
BMI: body mass index
CRP: C-reactive protein
FNDC5: fibronectin type III domain-containing protein 5
GLUT: glucose transporter
eGFR: estimated glomerular filtration rate
HDL: high-density lipoprotein
HOMA-IR: homeostasis model assessment of insulin resistance
IFG: impaired fasting glucose
LDL: low-density lipoprotein
MDRD: modification of diet in renal disease
NCEP: national cholesterol education program
PGC1α: peroxisome proliferator-activated receptor-γ coactivator 1α
SMI: skeletal muscle mass index
SD: standard deviation
SBP: systolic blood pressure
VIF: variance inflation factor
