# Supplementary material for: Increase in relative skeletal muscle mass over time and its inverse association with metabolic syndrome development: a 7-year retrospective cohort study
Source: Cardiovasc Diabetol. 2018 Feb 5;17:23. doi: 10.1186/s12933-018-0659-2 (PMC5798183; doi:10.1186/s12933-018-0659-2)
Supplement: Supplementary file 1 — Additional file 1: Table S1. Association between baseline sex-specific ASM/BMI tertiles and incidence of metabolic syndrome (Cox model) (N = 14,830). [file 12933_2018_659_MOESM1_ESM.docx]

**Table S1 Association between baseline sex-specific ASM/BMI tertiles and incidence of metabolic syndrome (Cox model) (N = 14,830)**

| ASM/BMI (m^2^)  Men  Women | Lowest tertile  (*n* = 4,943)  0.768 (0.133)  0.870 (0.068)  0.631 (0.051) | | Middle tertile  (*n* = 4,944)  0.856 (0.132)  0.960 (0.062)  0.717 (0.042) | | | Highest tertile  (*n* = 4,943)  0.952 (0.145)  1.059 (0.083)  0.810 (0.067) | | |  |
| --- | --- | --- | --- | --- | --- | --- | --- | --- | --- |
|  | | Referent | HR | 95% CI | *P* value | HR | 95% CI | *P* value | *P* for trend |
| Model 1 | | 1 | 0.72 | 0.66, 0.78 | <0.001 | 0.48 | 0.43, 0.52 | <0.001 | <0.001 |
| Model 2 | | 1 | 0.76 | 0.70, 0.83 | <0.001 | 0.53 | 0.48, 0.58 | <0.001 | <0.001 |
| Model 3 | | 1 | 0.88 | 0.81, 0.96 | 0.003 | 0.70 | 0.64, 0.77 | <0.001 | <0.001 |
| Model 4 | | 1 | 0.89 | 0.81, 0.96 | 0.005 | 0.71 | 0.64, 0.78 | <0.001 | <0.001 |

Model 1: crude.

Model 2: Model 1+ further adjusted for age.

Model 3: Model 2 + further adjusted for waist circumference.

Model 4: Model 3 + further adjusted for family history of diabetes, smoking status, regular exercise, eGFR, and CRP.

*ASM* appendicular skeletal muscle mass, *BMI* body mass index*, CI* confidence interval, *CRP* C-reactive protein, *eGFR* estimated glomerular filtration rate, *HR* hazard ratio.
