# Supplementary material for: Increase in relative skeletal muscle mass over time and its inverse association with metabolic syndrome development: a 7-year retrospective cohort study
Source: Cardiovasc Diabetol. 2018 Feb 5;17:23. doi: 10.1186/s12933-018-0659-2 (PMC5798183; doi:10.1186/s12933-018-0659-2)
Supplement: Supplementary file 2 — Additional file 2: Table S2. Association between baseline sex-specific SMI tertiles and incidence of metabolic syndrome in men (N = 8476) and women (N = 6354). [file 12933_2018_659_MOESM2_ESM.docx]

**Table S2 Association between baseline sex-specific SMI tertiles and incidence of metabolic syndrome in men (N = 8,476) and women (N = 6,354)**

| *n*=14,830 | Lowest tertile  (*n* = 4,943) | Middle tertile  (*n* = 4,944) | | | Highest tertile  (*n* = 4,943) | | |  |
| --- | --- | --- | --- | --- | --- | --- | --- | --- |
|  | Referent | HR | 95% CI | *P* value | HR | 95% CI | *P* value | *P* for trend |
|  |  |  |  |  |  | P for interaction = 0.923 | | |
| Men  (*n* = 8,476) | Lowest tertile  (*n* = 2,825) | Middle tertile  (*n* = 2,826) | | | Highest tertile  (*n* = 2,825) | | | |
| Model 1 | 1 | 0.60 | 0.55, 0.66 | <0.001 | 0.28 | 0.25, 0.32 | <0.001 | <0.001 |
| Model 2 | 1 | 0.60 | 0.55, 0.67 | <0.001 | 0.29 | 0.25, 0.32 | <0.001 | <0.001 |
| Model 3 | 1 | 0.91 | 0.82, 1.01 | 0.081 | 0.65 | 0.56, 0.74 | <0.001 | <0.001 |
| Model 4 | 1 | 0.91 | 0.82, 1.01 | 0.088 | 0.65 | 0.56, 0.75 | <0.001 | <0.001 |
| Women  (*n* = 6,354) | Lowest tertile  (*n* = 2,118) | Middle tertile  (*n* = 2,118) | | | Highest tertile  (*n* = 2,118) | | | |
| Model 1 | 1 | 0.48 | 0.41, 0.56 | <0.001 | 0.21 | 0.17, 0.25 | <0.001 | <0.001 |
| Model 2 | 1 | 0.55 | 0.48, 0.64 | <0.001 | 0.27 | 0.22, 0.33 | <0.001 | <0.001 |
| Model 3 | 1 | 0.86 | 0.73, 1.02 | 0.077 | 0.55 | 0.44, 0.70 | <0.001 | <0.001 |
| Model 4 | 1 | 0.87 | 0.73, 1.03 | 0.099 | 0.56 | 0.44, 0.70 | <0.001 | <0.001 |

Model 1: crude.

Model 2: Model 1+ further adjusted for age.

Model 3: Model 2 + further adjusted for BMI.

Model 4: Model 3 + further adjusted for family history of diabetes, smoking status, regular exercise, eGFR, and CRP.

*BMI* body mass index, *CI* confidence interval, *CRP* C–reactive protein, *eGFR* estimated glomerular filtration, *HR* hazard ratio.
