# Supplementary material for: Increase in relative skeletal muscle mass over time and its inverse association with metabolic syndrome development: a 7-year retrospective cohort study
Source: Cardiovasc Diabetol. 2018 Feb 5;17:23. doi: 10.1186/s12933-018-0659-2 (PMC5798183; doi:10.1186/s12933-018-0659-2)
Supplement: Supplementary file 3 — Additional file 3: Table S3. Correlations between changes of skeletal muscle mass index and glycometabolic parameters. [file 12933_2018_659_MOESM3_ESM.docx]

**Table S3 Correlations between changes of skeletal muscle mass index and glycometabolic parameters.**

| Variable | ΔSMI (%) | |
| --- | --- | --- |
|  | R | *P* value |
| ΔWaist circumference (cm) | -0.209 | <0.001 |
| ΔBody weight (kg) | -0.494 | <0.001 |
| ΔBMI (kg/m^2^) | -0.586 | <0.001 |
| ΔASM (kg) | 0.588 | <0.001 |
| ΔFat mass (kg) | -0.719 | <0.001 |
| ΔPercent fat mass (%) | -0.834 | <0.001 |
| ΔSBP (mmHg) | -0.124 | <0.001 |
| ΔDBP (mmHg) | -0.146 | <0.001 |
| ΔHbA_1c_ (%) | -0.063 | <0.001 |
| ΔFasting glucose (mg/dL) | -0.092 | <0.001 |
| ΔFasting insulin (μIU/mL) | -0.094 | <0.001 |
| ΔHOMA-IR | -0.111 | <0.001 |
| ΔTotal cholesterol (mg/dL) | -0.097 | <0.001 |
| ΔTriglycerides (mg/dL) | -0.126 | <0.001 |
| ΔHDL cholesterol (mg/dL) | 0.074 | <0.001 |
| ΔLDL cholesterol (mg/dL) | -0.150 | <0.001 |
| ΔC-reactive protein (mg/L) | -0.012 | 0.186 |

*ASM* appendicular skeletal muscle mass, *BMI* body mass index, *DBP* diastolic blood pressure, *HDL* high density lipoprotein, *HOMA–IR* Homeostasis model assessment of insulin resistance, *LDL* low density lipoprotein, *SBP* systolic blood pressure, *SMI* skeletal muscle mass index.

ΔThe differences of the variables for the 1-year interval from baseline.
