# Supplementary material for: Increase in relative skeletal muscle mass over time and its inverse association with metabolic syndrome development: a 7-year retrospective cohort study
Source: Cardiovasc Diabetol. 2018 Feb 5;17:23. doi: 10.1186/s12933-018-0659-2 (PMC5798183; doi:10.1186/s12933-018-0659-2)
Supplement: Supplementary file 4 — Additional file 4: Table S4. Association between continuous variable of change in skeletal muscle mass index from baseline to year 1 and incidence of metabolic syndrome (Cox model). [file 12933_2018_659_MOESM4_ESM.docx]

**Table S4** **Association between continuous variable of change in SMI from baseline to year 1 and incidence of metabolic syndrome (Cox model)**

|  | HR | 95% CI | *P* value |
| --- | --- | --- | --- |
| Change in SMI from baseline over 1 year (%) | 0.89 | 0.85, 0.94 | <0.001 |
| Sex (0=male, 1=female) | 0.66 | 0.53, 0.82 | <0.001 |
| Age (year) | 1.02 | 1.01, 1.02 | <0.001 |
| BMI (kg/m^2^) | 1.22 | 1.19, 1.25 | <0.001 |
| Family history of diabetes | 1.09 | 0.92, 1.30 | 0.326 |
| Smoking status |  |  |  |
| Never | Referent |  |  |
| Past | 1.02 | 0.89, 1.17 | 0.788 |
| Current | 1.48 | 1.27, 1.72 | <0.001 |
| Regular exercise | 1.08 | 0.93, 1.25 | 0.298 |
| eGFR (ml/min/1.73m^2^) | 0.99 | 0.99, 1.00 | 0.020 |
| C-reactive protein (mg/L) | 1.13 | 1.02, 1.26 | 0.016 |
| Baseline SMI (%) | 0.90 | 0.88, 0.93 | <0.001 |

*BMI* body mass index, *CI* confidence interval, *CRP* C–reactive protein, *eGFR* estimated glomerular filtration, *HOMA–IR* Homeostasis model assessment of insulin resistance, *HR* hazard ratio, *SMI* skeletal muscle mass index.
