# Supplementary material for: Increase in relative skeletal muscle mass over time and its inverse association with metabolic syndrome development: a 7-year retrospective cohort study
Source: Cardiovasc Diabetol. 2018 Feb 5;17:23. doi: 10.1186/s12933-018-0659-2 (PMC5798183; doi:10.1186/s12933-018-0659-2)
Supplement: Supplementary file 5 — Additional file 5: Table S5. Association between change in ASM/BMI index over 1 year and incidence of metabolic syndrome (Cox model) (N = 11,639). [file 12933_2018_659_MOESM5_ESM.docx]

**Table S5 Association between tertiles of change in ASM/BMI index over 1 year and incidence of metabolic syndrome (Cox model) (N = 11,639)**

| ASM/BMI 1-year increases (m^2^) | Lowest tertile  (*n* = 3,879)  -0.037 (0.019) | | Middle tertile  (*n* = 3,880)  -0.004 (0.007) | | | Highest tertile  (*n* = 3,880)  0.030 (0.021) | | |  |
| --- | --- | --- | --- | --- | --- | --- | --- | --- | --- |
|  | | Referent | HR | 95% CI | *P* value | HR | 95% CI | *P* value | *P* for trend |
| Model 1 | | 1 | 1.06 | 0.94, 1.18 | 0.341 | 0.90 | 0.80, 1.01 | 0.079 | 0.027 |
| Model 2 | | 1 | 1.11 | 0.99, 1.24 | 0.085 | 0.96 | 0.85, 1.09 | 0.559 | 0.062 |
| Model 3 | | 1 | 1.02 | 0.91, 1.14 | 0.735 | 0.88 | 0.78, 0.99 | 0.034 | 0.032 |
| Model 4 | | 1 | 1.02 | 0.91, 1.14 | 0.745 | 0.87 | 0.77, 0.99 | 0.028 | 0.026 |
| Model 5 | | 1 | 0.99 | 0.88, 1.11 | 0.829 | 0.84 | 0.75, 0.95 | 0.007 | 0.011 |

Model 1: crude.

Model 2: Model 1+ further adjusted for sex and age.

Model 3: Model 2 + further adjusted for waist circumference

Model 4: Model 3 + further adjusted for family history of diabetes, smoking status, regular exercise, eGFR, and CRP.

Model 5: Model 4 + further adjusted for baseline ASM/BMI index.

*ASM* appendicular skeletal muscle mass, *BMI* body mass index, *CI* confidence interval, *CRP* C-reactive protein, *eGFR* estimated glomerular filtration rate, *HR* hazard ratio.
