# Supplementary material for: Increase in relative skeletal muscle mass over time and its inverse association with metabolic syndrome development: a 7-year retrospective cohort study
Source: Cardiovasc Diabetol. 2018 Feb 5;17:23. doi: 10.1186/s12933-018-0659-2 (PMC5798183; doi:10.1186/s12933-018-0659-2)
Supplement: Supplementary file 6 — Additional file 6: Table S6. Association between continuous variable of change in ASM/BMI index from baseline to year 1 and incidence of metabolic syndrome (Cox model). [file 12933_2018_659_MOESM6_ESM.docx]

**Table S6** **Association between continuous variable of change in ASM/BMI from baseline to year 1 and incidence of metabolic syndrome (Cox model)**

|  | HR | 95% CI | *P* value |
| --- | --- | --- | --- |
| Change in ASM/BMI index from baseline over 1 year (per 0.10 m^2^) | 0.75 | 0.64, 0.88 | <0.001 |
| Sex (0=male, 1=female) | 1.35 | 1.09, 1.66 | 0.006 |
| Age (year) | 1.01 | 1.00, 1.01 | 0.136 |
| Waist circumference (cm) | 1.10 | 1.09, 1.11 | <0.001 |
| Family history of diabetes | 1.09 | 0.92, 1.30 | 0.327 |
| Smoking status |  |  |  |
| Never | Referent |  |  |
| Past | 0.98 | 0.85, 1.12 | 0.729 |
| Current | 1.38 | 1.19, 1.61 | <0.001 |
| Regular exercise | 1.05 | 0.90, 1.21 | 0.550 |
| eGFR (ml/min/1.73m^2^) | 0.99 | 0.99, 1.00 | 0.005 |
| C-reactive protein (mg/L) | 1.13 | 1.01, 1.26 | 0.030 |
| Baseline ASM/BMI index (per 0.10 m^2^) | 0.87 | 0.82, 0.92 | <0.001 |

*ASM* Appendicular skeletal muscle mass*, BMI* body mass index, *CI* confidence interval, *eGFR* estimated glomerular filtration, *HR* hazard ratio.
