# Supplementary material for: Increase in relative skeletal muscle mass over time and its inverse association with metabolic syndrome development: a 7-year retrospective cohort study
Source: Cardiovasc Diabetol. 2018 Feb 5;17:23. doi: 10.1186/s12933-018-0659-2 (PMC5798183; doi:10.1186/s12933-018-0659-2)
Supplement: Supplementary file 7 — Additional file 7: Table S7. Association between change in SMI over 1 year and incidence of metabolic syndrome in men (N = 6746) and women (N = 4893). [file 12933_2018_659_MOESM7_ESM.docx]

**Table S7 Association between change in SMI over 1 year and incidence of metabolic syndrome in men (*n* = 6,746) and women (*n* = 4,893)**

| *n* = 11,639 | Group 1  (< 0%)  (*n* =6,525) | Group 2  (0-1%)  (*n* = 3,734) | | | Group 3  (>1%)  (*n* = 1,380) | | |  |
| --- | --- | --- | --- | --- | --- | --- | --- | --- |
|  | Referent | HR | 95% CI | *P* value | HR | 95% CI | *P* value | *P* for trend |
|  |  |  |  |  |  | P for interaction = 0.506 | | |
| Men  (*n* = 6,746) | Group 1  (< 0%)  (*n* =4,081) | Group 2  (0-1%)  (*n* = 2,010) | | | Group 3  (>1%)  (*n* = 655) | | |  |
| Model 1 | 1 | 0.90 | 0.79, 1.03 | 0.113 | 0.71 | 0.57, 0.89 | 0.003 | 0.008 |
| Model 2 | 1 | 0.90 | 0.79, 1.03 | 0.116 | 0.72 | 0.57, 0.90 | 0.004 | 0.009 |
| Model 3 | 1 | 0.86 | 0.76, 0.98 | 0.025 | 0.67 | 0.54, 0.84 | 0.001 | 0.001 |
| Model 4 | 1 | 0.87 | 0.76, 0.99 | 0.032 | 0.67 | 0.53, 0.84 | 0.001 | 0.001 |
| Model 5 | 1 | 0.83 | 0.73, 0.94 | 0.005 | 0.63 | 0.50, 0.79 | <0.001 | <0.001 |
| Women  (*n* = 4,893) | Group 1  (< 0%)  (*n* =2,444) | Group 2  (0-1%)  (*n* = 1,724) | | | Group 3  (>1%)  (*n* = 725) | | |  |
| Model 1 | 1 | 1.04 | 0.86, 1.25 | 0.717 | 0.88 | 0.66, 1.17 | 0.372 | 0.551 |
| Model 2 | 1 | 1.06 | 0.88, 1.28 | 0.550 | 0.93 | 0.70, 1.24 | 0.616 | 0.659 |
| Model 3 | 1 | 1.01 | 0.84, 1.22 | 0.917 | 0.81 | 0.61, 1.08 | 0.153 | 0.315 |
| Model 4 | 1 | 0.99 | 0.82, 1.20 | 0.939 | 0.82 | 0.61, 1.09 | 0.163 | 0.360 |
| Model 5 | 1 | 0.95 | 0.79, 1.16 | 0.631 | 0.78 | 0.58, 1.04 | 0.085 | 0.226 |

Model 1: crude.

Model 2: Model 1+ further adjusted for age.

Model 3: Model 2 + further adjusted for BMI.

Model 4: Model 3 + further adjusted for family history of diabetes, smoking status, regular exercise, eGFR, and CRP.

Model 5: Model 4 + further adjusted for baseline SMI.

*BMI* body mass index, *CI* confidence interval, *CRP* C–reactive protein, *eGFR* estimated glomerular filtration, *HR* hazard ratio.
